# PaleoClim, high spatial resolution paleoclimate surfaces for global land areas

**DOI:** 10.1038/sdata.2018.254

**Published:** 2018-11-13

**Authors:** Jason L. Brown, Daniel J. Hill, Aisling M. Dolan, Ana C. Carnaval, Alan M. Haywood

**Affiliations:** 1 Cooperative Wildlife Research Laboratory & The Center for Ecology, Southern Illinois University, Carbondale, IL 62901, USA; 2 School of Earth and Environment, University of Leeds, Leeds, LS2 9JT, UK; 3 City College of New York and The Graduate Center, City University of New York, New York, NY 10031, USA

**Keywords:** Palaeoclimate, Ecological modelling

## Abstract

High-resolution, easily accessible paleoclimate data are essential for environmental, evolutionary, and ecological studies. The availability of bioclimatic layers derived from climatic simulations representing conditions of the Late Pleistocene and Holocene has revolutionized the study of species responses to Late Quaternary climate change. Yet, integrative studies of the impacts of climate change in the Early Pleistocene and Pliocene – periods in which recent speciation events are known to concentrate – have been hindered by the limited availability of downloadable, user-friendly climatic descriptors. Here we present PaleoClim, a free database of downscaled paleoclimate outputs at 2.5-minute resolution (~5 km at equator) that includes surface temperature and precipitation estimates from snapshot-style climate model simulations using HadCM3, a version of the UK Met Office Hadley Centre General Circulation Model. As of now, the database contains climatic data for three key time periods spanning from 3.3 to 0.787 million years ago: the Marine Isotope Stage 19 (MIS19) in the Pleistocene (~787 ka), the mid-Pliocene Warm Period (~3.264–3.025 Ma), and MIS M2 in the Late Pliocene (~3.3 Ma).

## Background & Summary

Shifts in climate and habitats have key evolutionary and ecological consequences, and are closely associated with contemporary biodiversity patterns^1,2^. Over the past decade, hundreds of studies have combined data on species occurrences with climate descriptions from interpolated weather-stations to model the distribution of animals and plants worldwide^3^. When projected into paleo- and future-climatic scenarios, these models are widely used to investigate the historic and future distributions of biodiversity^4–7^. However, the limited availability of easily accessible climate data for time periods other than the mid-Holocene (6 Ka), the Last Glacial Maximum (LGM, 21 Ka) and the Last Interglacial (130 Ka) has posed a significant impediment to scientists interested in biological responses to past climate change^8,9^.

Traditionally, those target periods have been the focus of biological investigations largely because they correspond to times of temperature extremes in the northern latitudes^1,10^. While patterns of climate change between those periods are shown to play a role in biodiversity patterns, there exists considerable spatial variation in their ability to explain the distribution of biological diversity in species-rich and threatened tropical areas^4,11–16^. Despite the utility of the paleoclimatologies spanning the last 130 Ka, a major impediment to ecological and evolutionary studies is the lack of easily accessible, high spatial resolution paleoclimatic data in a format directly compatible with most GIS software, particularly those describing earlier time periods.

**Figure 1:**
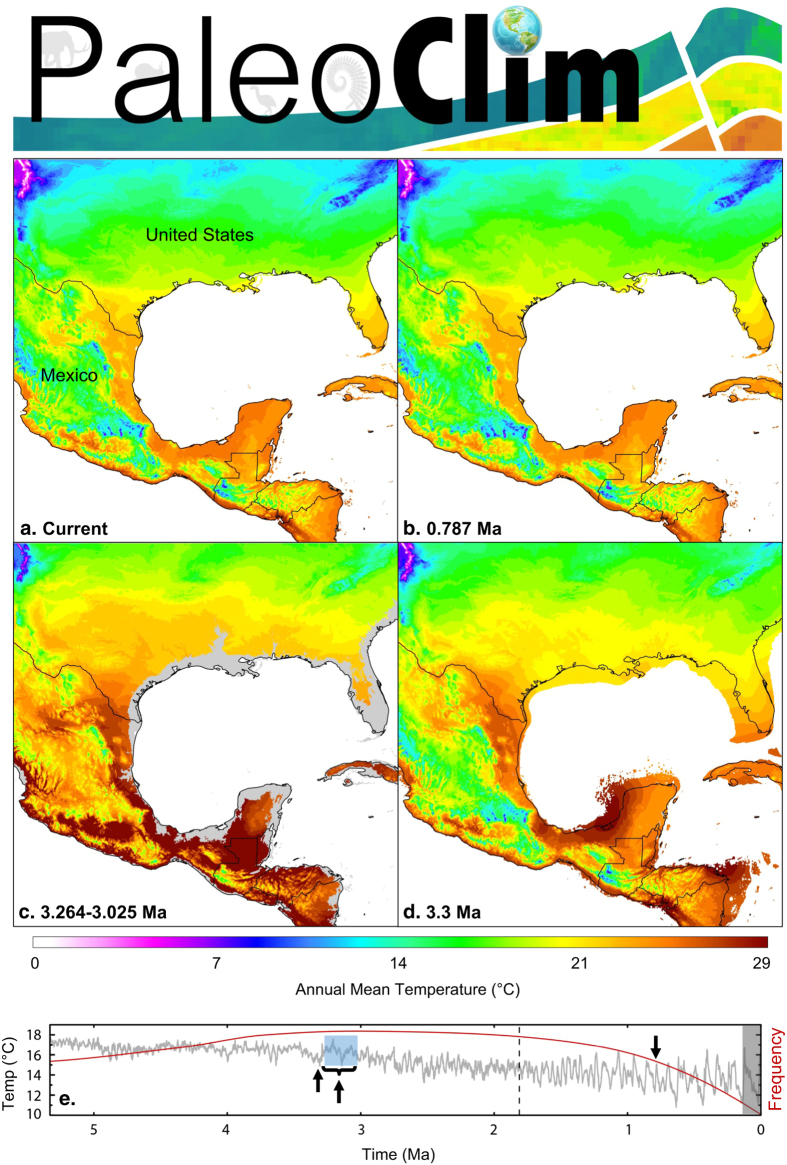
Paleoclim datasets. (**a**) Current climate (from CHELSA). (**b**) Pleistocene MIS19 (ca. 787 Ka). (**c**) mid-Pliocene Warming Period (3.265-3.025 Ma). Sea-levels were on average 25 m higher than modern times (depicted in gray). The grayed areas are not part of the final corresponding datasets. (**d**) Pliocene M2 period (3.3 Ma). Sea-level is 40 m lower than current levels and, in many areas, coastlines were expanded. (**e**) Sea-surface temperature changes (left axis) and speciation rates (right axis) during the last 5 Ma (gray line and red line, respectively. Data from^17, 54^). Black arrows highlight time periods of this study. The gray box depicts the time periods of high-resolution climate data currently widely available to biologists.

To fill this gap, and given that the most common divergence times between extant sister species have been placed at 1–4 Ma, with relatively few divergence times spanning the last 130 kyrs^17,18^, we have developed PaleoClim. Our aim is to provide the scientific community with data more reflective of the time periods under which speciation occurs, allowing for a more complete understanding of the drivers of biodiversity processes and patterns. PaleoClim is a free database of downscaled paleomodels at 2.5 arc-minute resolution (~5 km at equator), representing temperature and precipitation estimates output from individual snapshot coupled atmosphere-ocean general circulation models from the Hadley Centre Coupled Model Version 3 (HadCM3). Building from these estimates, we have derived paleo bioclimatic layers that represent annual averages (e.g. mean annual temperature, annual precipitation), seasonality (e.g. annual range in temperature and precipitation), and extreme or limiting environmental factors (e.g. temperature of the coldest and warmest month, and precipitation of the wet and dry quarters), akin to WorldClim^19^. To date, the database contains high-resolution terrestrial data for three key periods: Marine Isotope Stage 19 (MIS19) in the Pleistocene (ca. 787 Ka), the mid-Pliocene Warm Period (mPWP, ca. 3.264-3.025 Ma) and Marine Isotope Stage M2 (M2), a glacial interval in the Late Pliocene (ca. 3.3 Ma, Fig. 1).

## Methods

### Paleoclimate Simulations

The paleoclimate simulations used here come from the HadCM3 version of the UK Met Office Unified Model General Circulation Model (GCM). This is a well-established coupled ocean atmosphere climate model, having contributed to the last three Intergovernmental Panel on Climate Change (IPCC) Assessment Reports (AR3, AR4 and AR5), and used to simulate climate for nearly 20 years^20,21^. HadCM3 has a horizontal resolution of 2.5° in latitude and 3.75° in longitude, and a higher resolution ocean of 1.25°×1.25° regular long-lat grid, with 19 vertical levels in atmosphere and 20 in the ocean. The atmospheric component has a time-step of 30 min, and is coupled to the ocean every day. Typically, the climatology is output every month, and the mean annual and monthly climate are calculated from these data. As the name GCM suggests, this class of climate model is able to reproduce the major circulations in the both the atmosphere and ocean, as well as major drivers of inter-annual variability^22^. The resolution also allows for synoptic weather patterns to be simulated, along with key climate oscillations, but may not simulate well local extremes or regions with high gradients (e.g. extreme convective events^23^). HadCM3 is in the middle of the range of overall climate sensitivities exhibited by the IPCC-class climate models^24,25^.

The paleoclimate simulations presented here (Table 1) are intended as an example of what is possible with the techniques employed, and represent significantly different time periods from what is currently broadly available to biologists. Ice cores provide the best possible constraints on past greenhouse gases beyond the instrumental record. This means that paleoclimate simulations of the last 800,000 years have a distinct advantage over those from previous time periods. Marine isotope stage 19 (MIS19) occurs at roughly 787 Ka and is the oldest Pleistocene interglacial covered by the latest EPICA Antarctic ice core^26^. This allows us to use well-constrained greenhouse gas concentrations of CO_2_^26^, CH_4_^27^ and N_2_O^28^, as well as accurate orbital parameters^29^. Prior to 400,000 years ago and MIS11, there are significant differences in the magnitude of glacial-interglacial cycles, both in the greenhouse gas concentrations and temperature responses. However, MIS19 has the most Holocene-like greenhouse gases and ice core temperature proxies of the interglacials that occur between 800,000 and 400,000 years ago. Further paleogeographic boundary condition change must have occurred over these timescales, but as there is no reconstruction currently available, the remaining boundary conditions have been kept as in the pre-industrial simulation.

The mid-Pliocene Warm Period (mPWP) simulation follows the PlioMIP protocols^25^, is a continuation of the original HadCM3 PlioMIP simulation^30^, and has been previously published by Hill^31^. The simulation has elevated atmospheric carbon dioxide concentrations set to 405 parts per million by volume (ppmv), reduced ice sheets^32^, a Piacenzian vegetation reconstruction^33^ and altered topography, particularly over the Rockies, Andes and East African Rift system^34^. As the underlying datasets are not the reconstruction of a specific point in time, but of the environmental conditions typical of a mid-Pliocene warm peak^35^, a modern orbit is given to the PlioMIP simulations.

The marine isotope stage M2 glacial period is the strongest Pliocene oxygen isotope excursion prior to the beginning of the Plio-Pleistocene transition, which marks the start of the Pleistocene glacial-interglacial cycles^36^. The magnitude of the oxygen isotope excursion suggests that large ice masses may have covered the Northern Hemisphere for a short time, although the exact locations of these ice sheets remains uncertain^37,38^. The ice sheets used in the simulations are based on the ice sheets of 116 Ka, which are the 40 m sea level rise volume equivalents from the last glacial cycle^37^. A reduction in atmospheric CO_2_ concentration to 220 ppmv^39^ was implemented in the climate model alongside orbital forcing appropriate for 3.3 Ma, although this is close to the modern orbital configuration^24^.

### Downscaling

We employed the Change-Factor method^19,40,41^ to downscale the paleoclimatic climatologies. This approach creates high-resolution layers by quantifying the differences between the paleo and current (control) climatologies for each raw variable, at the native model-specific spatial resolution. This functions as a calibration step to measure the raw climate anomalies at the coarser spatial scale climate model. Once this step is completed, the difference layers (commonly called delta layers, change-factor differences, or climate change anomalies) are downscaled to high-resolutions (typically 1–20 km) and summed to a matching high-resolution current climate variable. This method is relatively quick, requiring less than a day of computational time per raster layer, and can be efficiently applied to global datasets. A major benefit of the Change-Factor method relative to other methods of downscaling is its ability to incorporate small-scale topographic nuances in regional climatologies that are often not captured in climate models, but present in the high-resolution current datasets. Examples include climatic differences in mountainous regions such as differences between valleys, mid-elevation ranges, and their peaks.

Here, we created global delta layers by subtracting the raw temperature and precipitation values of each snapshot paleoclimatic simulation from corresponding HadCM3 control simulations that represent the pre-industrial era. The delta layer represents the pixel-by-pixel changes from pre-industrial conditions, within the constraints of each snapshot climate simulation. The delta layers were downscaled 60-fold from 2.5 arc-degrees to 2.5 arc-minutes (ca. 5 km) using a tensioned spline in ArcGIS 10.5 (sampling = 12 nearest observations to a focal point, weight of 0.1, ESRI 2018). A spline is a deterministic interpolation method that has been shown to deliver similar results when compared to kriging^41–46^, and it has been commonly considered as appropriate for interpolation environmental variables^44^. We used a tensioned spline (instead of a regularized spline) to avoid extraneous inflection points, and more generally to preserve shape properties, such as monotonicity and convexity, of a set of data points - and to do so without sacrificing smoothness^47^. Spline approaches are based on requirement that the interpolation function passes through the data points, but also yield the smoothest transition as possible.

The high-resolution delta layers were then summed to a corresponding current monthly temperature or precipitation climate layers from the Climatologies at High-Resolution for the Earth’s Land Surface Areas (CHELSA) database^48^ at the same resolution. Though rare in our analyses, negative precipitation values were converted to zero. To reduce pixel-depth and file sizes of final products, all monthly temperature raster layers were multiplied by 10 and converted to integers. Prior to the creation of bioclimate layers, final monthly layers were adjusted to the mean sea-level of paleoclimatic period, based on adjustments to a contemporary bathymetry dataset^49^.

We also explored the use of ratios of anomalies (ROAs), instead of raw differences, to downscale precipitation. A major caveat to the Change-Factor method regards transferring the generalized spatial patterns in the climate model simulations to the regional mosaic of habitats in the high-resolution climates, as the model predicted climate patterns are uniformly applied to the latter. Other studies using the Change-Factor approach have advocated the use of ROAs to the corresponding baseline conditions for downscaling precipitation (vs. raw differences used here, and for temperatures universally elsewhere). Those studies argue that the use of the raw difference method^40^ may result in inaccurate inferences in regions of strong rainfall gradients, and state that ROAs are more robust to maintaining original patterns in downscaling when managing larger values^40,41^.

### Topographic Differences

At a coarse level, all paleoclimatic layers account for topographic shifts incurred between now and the past. For instance, the global topography used in the Pliocene simulations, derived from the Pliocene Research, Interpretation and Synoptic Mapping (PRISM3) dataset, are largely, but not entirely, similar to the modern topography^32^. Notable exceptions include: 1) the mountains of the western Cordillera of western North America and the Andean mountains in South America, which were then, in a few regions, lower than modern altitudes^50,51^, 2) the elevation of some of the regions now covered by the Greenland and Antarctica Ice Sheets, which then experienced a net decrease caused by a reduction in the size of the ice sheets themselves, 3) the east African rift zone, which then reached higher elevations than at present, as indicated in the literature^50,51^. All topographic changes were incorporated into HadCM3, and the simulated climates are reflective of those differences. However, because we downscaled the final datasets with modern climatologies, results in these particular areas should be carefully evaluated.

Unlike for the mid-Pliocene simulation, the M2 glacial climate simulation only has changes to topography resulting from changes in the ice sheets. Over Antarctica, Greenland and North America, changes over the ice sheet regions generally led to uplift in surface topography of between 50 and 500 meters compared with present day, but glacioisostatic rebound leads to reductions in the topography of neighbouring regions^37^. Regions outside those impacted by ice sheets were kept at modern topography. The MIS19 simulation use identical topography to the pre-industrial simulation.

### Bioclimatic parameters

From the high-resolution monthly temperature and precipitation values, we calculated a set of derived parameters broadly used in ecological applications. These bioclimatic variables are derived from the monthly mean temperature (or minimum and maximum temperature, depending on their availability) and precipitation values. They are specifically developed for species distribution modelling and related ecological applications (see Table 2 for a list and common nomenclature). For some paleo simulations (e.g. mPWP), the monthly maximum and minimum temperatures were not available. In these instances, the bioclimatic layers that represent annual averages (mean annual temperature, annual precipitation), seasonality (annual range in temperature and precipitation), and extreme or limiting environmental factors (temperature of the coldest and warmest quarters, and precipitation of the wet and dry quarters) could not be created (Bio_2, Bio_3, Bio_5, Bio_6, and Bio_7). In this transformation, a quarter is defined as the period of three months (1/4 of the year). Output bioclimate layers were saved as individual GeoTiffs (*tif) and projected in the WGS 1984 projection.

## Data Records

The Paleoclim data depict records for monthly mean temperature in °C and precipitation values in mm/month, and derived bioclimatic variables for a 30-year simulation period in the form of GeoTIFF files. The high resolution paleoclimatic bioclimatic variables and the original raw HadCM3 GCM monthly paleoclimate variables are freely available at Figshare (Data Citation 1). The latest versions of paleoclimate bioclimatic data are also freely available at http://www.paleoclim.org. See Table 2 for naming conventions and specific details for each provided variable.

### Code availability

The procedure for generating bioclimatic variables followed WorldClim^19^ and used the ‘biovars’ function of the R package *dismo*^52^ (see Supplementary File 1 for the code used in this study).

## Technical Validation

### Downscaling

We carefully explored the use of raw differences vs. ratios of anomalies to calibrate downscaled precipitation data under the Change Factor method. Understanding how the ‘raw difference’ and ROA calibration methods can dramatically change output paleoclimatic patterns is straightforward. Imagine an observed rainfall at a specific location to be 2.0 m/mo and 1.0 m/mo in the paleo- and current simulations, respectively. In this situation, the raw difference of precipitation is +1 m, while ROA is 2. Now let us assume that the precipitation values in the high-resolution modern dataset used to downscale the delta layers ranges, within that same area, from 0.25 m to 4.0 m. The raw difference method would yield paleoclimatic rainfall estimates, in the corresponding high-resolution dataset, that range from 1.25–5.0 m. In contrast, the ROA method would yield values ranging from 0.5–8.0 m, a much wider interval. It is important to also point out that this example oversimplifies the Change-Factor process, because delta layers are downscaled prior to summation or multiplication, which results in intermediate values between input delta layer points and the final high-resolution values, accordingly.

**Figure 2:**
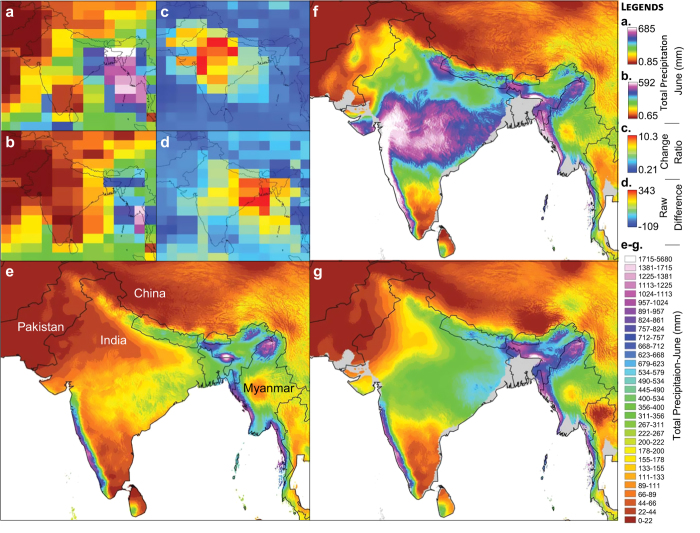
Comparison of Change factor calibration methods for precipitation data. (**a**) HadCM3 results for the mid-Pliocene Warm Period. (**b**) HadCM3 results for pre-industrial climates. (**c**) Ratios of anomaly calibration layer (a/b) (**d**) Raw difference calibration layer (b-a). (**e**) High-resolution total precipitation of June^48^ (contemporary times). (**f**) Mid-Pliocene Warm Period total precipitation in June using ratios of anomaly calibration. (**g**) Mid-Pliocene Warm Period total precipitation in June using raw difference calibration

When using raw differences in precipitation, we found no evidence of illogical transitions in our datasets in areas of high rainfall (Fig 2). In contrast, the use of ROA (vs. raw differences) resulted in inferior results (Fig. 2). For illustration purposes, we show the downscaled high-resolution layers of the mid-Pliocene Warm Period, in an area of a high range of rainfall, the region of the Himalayan Mountains (Fig 2). For the month of June, for instance, the use of the raw differences calibration method resulted in a total precipitation estimate ranging between 0 and 2.8 m/mo. The use of the ROA, in turn, yielded a much larger range, between 0 and 5.7 m/mo. For this same region, the raw values from the original paleoclimate simulation ranged from 0.85 to 0.69 m/mo, from 0.65 to 0.59 m/mo for the pre-industrial control simulations, and from 0 to 2.5 m for the current high-resolution layers. The resulting differences in the ROA-derived high-resolution paleolayer is hence over a 2-fold difference in maximum precipitation values.

Similarly, when the hi-resolution data are aggregated to 2.5 degrees (by calculating the mean value per area), and correlated to the raw Pliocene values from HadCM3, we observe a Pearson correlation coefficient of 0.810 in the raw change method and 0.667 in ratio of anomalies - for the same corresponding region and time. At a global level, we measured a Pearson correlation coefficient of 0.823 in the raw change method, and 0.612 in the ratio of anomalies. These results are also matched by the visualization of both high-resolution layers compared to the raw climate model values (Fig 2, comparing A & G vs. A. & F). Though this is just one example, these observations were consistent among all months evaluated and between different climate model simulations.

A second concern regards the extent by which areas of low current rainfall change as a result of ROA downscaling, particularly those with zero modern rainfall. When the modern value is zero, the downscaling values cannot change, as any number multiplied against zero will be zero. Therefore, when using the ROA method, areas of the eastern Sahara Desert, for instance, will never possess rainfall amounts above zero for many months, despite the fact we know this is historically inaccurate^53^, and potentially reflected in the climate model simulations. Furthermore, if the rainfall value is small in the current high-resolution dataset, values change only slightly when using ROA, even if the ROA value is high. Given the profound ecological impacts of precipitation in water-limited ecosystems, the two different calibration methods dramatically impact downscaled precipitation in these habitats. We recommend avoiding ROA in this case.

Overall, the use of ROAs (vs. raw differences) resulted in inferior paleoclimatic outputs due to multiplication of ratios in the delta layers against a high-resolution current climatology (vs. summing in the raw difference method). Hence, we suggest that users apply the raw difference method to precipitation data or utilize the raw difference-based paleoclimate outputs in their environmental, ecological, and evolutionary analyses. This approach is more sensitive to changes in low precipitation environments, more reflective of the raw paleoclimate values from HadCM3, and not as confounded by modern precipitation levels (i.e. areas with zero monthly precipitation). In the future releases, we plan to evaluate a hybrid approach that averages the outputs from both calibration methods.

## Usage Notes

When citing data from Paleoclim.org, please cite both this manuscript and the original manuscript(s) that generated to each climatology (see Table 1). This supports the continued generation of these derivative works, the primary research of the groups generating them, and promotes collaboration among PaleoClim and other researchers. PaleoClim reduces the amount of time that would be spent developing common solutions and provides data in a consistent nomenclature and common format, making data easier to use. We plan to regularly expand the PaleoClim database: providing additional paleoclimate time-periods, and new or improved GCMs of existing datasets with paleoclimate variability (vs. a mean simulation value). For questions, collaborative inquires, or suggestions regarding PaleoClim, go to our Google Group (https://groups.google.com/forum/#!forum/paleoclim) or email paleoclim@gmail.com. Data are feely available under the Creative Commons License: CC BY.

## Additional information

**How to cite this article**: Brown, J. L. *et al*. PaleoClim, high spatial resolution paleoclimate surfaces for global land areas. *Sci. Data*. 5:180254 doi: 10.1038/sdata.2018.254 (2018).

**Publisher’s note**: Springer Nature remains neutral with regard to jurisdictional claims in published maps and institutional affiliations.

## Supplementary Material



Supplementary File 1

## Figures and Tables

**Table 1:** Key parameters for the HadCM3 simulations currently in the Paleoclim database and presented here.

**Time Period**	**General boundary conditions**	**Orbital parameters**	**CO_2_ (ppmv)**	**CH_4_ (ppbv)**	**N_2_O (ppbv)**	**Sea level (above pre-industrial)**	**Citation**
MIS19	Pre-industrial	787 Ka	260.3	739	303.3	0 m	This study
mPWP	PlioMIP (Haywood et al., 2013)	Modern	405	760	270	+25 m	Hill, 2015^31^
M2	116 Ka (Singarayer & Valdes, 2010)	3.3 Ma	220	760	270	−40m	Dolan et al., 2015^37^

**Table 2:** Variables, units, and naming conventions.

**Variable name**	**Variable details**
Bio_1	Annual Mean Temperature [°C*10]
Bio_2	Mean Diurnal Range [°C]^ǂ^
Bio_3	Isothermality^ǂ^
Bio_4	Temperature Seasonality [standard deviation*100]
Bio_5	Max Temperature of Warmest Month [°C*10]^ǂ^
Bio_6	Min Temperature of Coldest Month [°C*10]^ǂ^
Bio_7	Temperature Annual Range [°C*10]^ǂ^
Bio_8	Mean Temperature of Wettest Quarter [°C*10]
Bio_9	Mean Temperature of Driest Quarter [°C*10]
Bio_10	Mean Temperature of Warmest Quarter [°C*10]
Bio_11	Mean Temperature of Coldest Quarter [°C*10]
Bio_12	Annual Precipitation [mm/year]
Bio_13	Precipitation of Wettest Month [mm/month]
Bio_14	Precipitation of Driest Month [mm/month]
Bio_15	Precipitation Seasonality [coefficient of variation]
Bio_16	Precipitation of Wettest Quarter [mm/quarter]
Bio_17	Precipitation of Driest Quarter [mm/quarter]
Bio_18	Precipitation of Warmest Quarter [mm/quarter]
Bio_19	Precipitation of Coldest Quarter [mm/quarter]
^ǂ^For some paleo simulations the monthly maximum and minimum temperatures were not available. In these instances Bio_2, Bio_3, Bio_5, Bio_6, Bio_7 could not be created.	
